# Association of age to nutritional status and muscle mass in children with transfusion-dependent *β*-thalassemia: a cross-sectional study

**DOI:** 10.3389/fnut.2024.1493502

**Published:** 2024-10-25

**Authors:** Li Wang, Luyang Zhang, Yanlan Yang, Yuan Luo, Lei Wang, Sandip Patil

**Affiliations:** ^1^Department of Clinical Nutrition, Shenzhen Children’s Hospital, Shenzhen, China; ^2^Department of Hematology and Oncology, Shenzhen Children’s Hospital, Shenzhen, China; ^3^Paediatric Research Institute Shenzhen Children’s Hospital, Shenzhen, China

**Keywords:** ***β***-thalassemia, transfusion-dependent ***β***-thalassemia, age, nutrition, muscles, children

## Abstract

**Background:**

Transfusion-dependent *β*-thalassemia (TDT) is a hereditary blood disorder that often leads to complications affecting growth, nutritional status, and muscle mass in children. This study aims to investigate the associations between age, nutritional status, and muscle mass in children with TDT, providing insights into the progressive impact of age on these parameters.

**Methods:**

One hundred twenty-two children with TDT from March 2023 to March 2024 were enrolled in this cross-sectional study. Their nutritional status was assessed using the 2006–2007 World Health Organization (WHO) Growth Charts, and their muscle mass was measured using bioelectrical impedance analysis (BIA). Data related to age, gender, weight, height, fat-free mass (FFM), skeletal muscle mass (SMM), and soft lean mass (SLM) of children were collected for comparative analysis from the hospital record room. Furthermore, Spearman’s rank correlation coefficients and regression analyses were utilized to investigate the associations between age and both nutritional status and muscle mass.

**Results:**

The results of this study revealed negative correlations between age and wasting (*r* = −0.26, *p* < 0.001), stunting (*r* = −0.28, *p* < 0.001), FFM (*r* = −0.3, *p* < 0.001), SMM (*r* = −0.23, *p* < 0.01), and SLM (*r* = −0.36, *p* < 0.001). The regression analysis indicated that age was an independent predictor of wasting, stunting, and reduced muscle mass in these children (all *p* < 0.001).

**Conclusion:**

Our study observed a trend of worsening stunting, wasting, and muscle loss in children with TDT as they age. These findings highlight the importance of monitoring both nutritional and muscular health in TDT patients. Early detection and comprehensive nutritional management may improve outcomes in this population.

## 1 Introduction

*β*-thalassemia is an inherited hemoglobinopathy characterized by reduced or absent *β*-globin chain synthesis, with transfusion-dependent β-thalassemia (TDT) representing the most severe phenotype on the clinical spectrum (1, 2). TDT is defined as a condition where patients require regular blood transfusions, typically every 2–4 weeks, to maintain adequate hemoglobin levels for normal growth and development (3). This severe form of *β*-thalassemia is predominantly found in the Mediterranean region, North Africa, the Sahara Desert, the Middle East, the Indian subcontinent, and Southeast Asia. In China, the highest prevalence of TDT is observed in 10 provinces south of the Yangtze River (4, 5). This geographical distribution highlights the global impact of TDT and the need for region-specific research. Patients with TDT necessitate lifelong, periodic blood transfusions and iron chelation therapy, subjecting them to distinctive health considerations, with particular implications for nutritional status and muscle mass composition (6, 7). Several studies have demonstrated that chronic anemia, iron overload, endocrine disorders, and the side effects of therapeutic interventions significantly impair the nutritional status and muscle mass in children with TDT (8). These factors may have cumulative and heterogeneous impacts as patients progress through different developmental stages. Nevertheless, studies elucidating the age-dependent associations with nutritional status and muscle mass metrics in this patient population remain limited. Research indicates that approximately 50% of pediatric TDT patients exhibit varying degrees of malnutrition, with stunting, wasting, and muscle atrophy being common manifestations (9, 10). Research shows that nutritional deficiencies, particularly related to muscle mass and growth, are more prevalent and severe in older children with TDT, with stunting and wasting rates increasing by approximately 10% per year (9, 11–13). However, research from other high-prevalence TDT regions, particularly East Asia, remains scarce, creating a significant knowledge gap that our study aim to address. Furthermore, children’s nutritional status is closely associated with their muscle mass and muscular strength (14). Studies on healthy pediatric populations have consistently demonstrated a positive correlation between age and both total body muscle mass and appendicular skeletal muscle mass index (15). Comparative studies indicate that children with TDT exhibit a significantly slower increase in muscle mass and bone mineral density, with difference observed in longitudinal studies comparing them to healthy peers (16). This observed disparity may be attributed to the potential nutritional deficiencies and reduced physical activity with TDT, resulting in skeletal muscle mass and function that fail to meet the expected levels for their age (17). In the context of TDT, where nutritional deficits are prevalent, elucidating the direct age-dependent associations with both nutritional status and muscle mass becomes crucial. This dual focus is essential for comprehensively understanding the complex interplay between disease progression, therapeutic interventions, and physiological development in pediatric TDT patients. Despite the clinical significance of these observations, there remains a paucity of research in this area, particularly in East Asian populations. To address these knowledge gaps, our study employs bioelectrical impedance analysis (BIA), a technique using electrical properties to analyze body composition. BIA offers several advantages, including its ability to detect malnutrition before changes in physical measurements and laboratory values become apparent (18, 19). The primary muscle mass indices measured by BIA include fat-free mass (FFM), soft lean mass (SLM), and skeletal muscle mass (SMM) (20). Using BIA provides a more accurate and non-invasive assessment of muscle mass in TDT children (21). Given the current gap in understanding, elucidating the relationships between age, nutritional status, and muscle mass in pediatric TDT patients is critical. This study aims to investigate the associations between age, nutritional status, and muscle mass in children with TDT, providing insights into the progressive impact of age on these parameters. By examining these relationships in an understudied population, we seek to enhance our comprehension of disease progression and address the current knowledge gap in TDT management for this specific demographic. We anticipate that our findings will contribute to improved clinical management protocols tailored to East Asian children with TDT, informing age-specific, personalized interventions. This research has the potential to lead to more targeted nutritional strategies and muscle mass preservation approaches, ultimately enhancing the quality of life for children with TDT.

## 2 Methods

In this study, a cross-sectional design was adopted to assess the nutritional status and muscle mass in children diagnosed with TDT. Children were enrolled upon admission to the hospital. As part of the standard admission protocol, nutritional risk screening was conducted, and measurements were taken for anthropometric indicators [height, weight, body mass index (BMI)] and body composition analyses [fat-free mass (FFM), skeletal muscle mass (SMM), and soft lean mass (SLM)].

### 2.1 Study objects

This study enrolled 122 TDT children in the Department of Hematology Oncology at Shenzhen Children’s Hospital from May 2023 to May 2024. Inclusion criteria inculded: (1) children who were diagnosed with TDT; (2) those aged ≥2 and ≤ 18 years; (3) children who were able to cooperate with the measurements of height, weight, and body composition analyses; and (4) children themselves and their guardians consented to participate in the study and signed the informed consent forms. Exclusion criteria were as follows: (1) Presence of severe pleural or peritoneal effusion; (2) children with cardiac pacemakers (BIA device manufacturer’s instructions); (3) Recent acute illness or hospitalization within the past month; and (4) Concurrent severe chronic diseases or conditions that might significantly affect nutritional status or body composition; and (5) TDT patients who have previously undergone hematopoietic stem cell transplantation (HSCT). TDT patients who have previously undergone HSCT were excluded because the intensive treatments associated with HSCT, such as high-dose chemotherapy, can significantly impact nutritional status and body composition (22) potentially confounding our study results. The study was conducted in accordance with the guide principles stipulated in the *Declaration of Helsinki* and with approval from the Ethics Committee of Shenzhen Children’s Hospital, reference number: 202313302 dated 2023/03/31, meeting the international ethical standards. According to the *Declaration of Helsinki*, all children were provided with informed consent forms before admission to agree on the use of their data for study.

### 2.2 Nutritional risk screening and assessment

The Screening Tool for the Assessment of Malnutrition in Pediatrics (STAMP) was utilized for nutritional risk screening. The STAMP comprises three sections: disease risk, nutritional intake, and growth condition. The nutritional risk outcomes were categorized as low risk (0–1 point), moderate risk (2–3 points), and high risk (≥4 points). Furthermore, the nutritional status of the children was assessed using the Z-score for growth standards and growth reference issued by the World Health Organization (WHO) (23, 24), with wasting and stunting indicators in seven levels: 3, 2, 1, 0, −1, −2, and-3.

### 2.3 Anthropometry

Anthropometric measurements include height and weight. Weight: The height and weight meter was placed against a wall on a flat ground and set to zero. Prior to measurement, the children were required to empty their bladder and bowels. The children should stand in the center of the scale platform stably, and the stable meter indication or the displayed figure should be recorded and filed, with an accuracy of 0.1 kg. Height: The children were required to stand barefoot on the platform, with arms hanging naturally at their sides, heels, buttocks, and shoulders touching the stadiometer post, and the head kept in a neutral position. The measurer adjusted the headpiece to contact the top of the head. The point where the leveled headpiece intersects with the scale is recorded and filed, with an accuracy of 0.1 cm.

### 2.4 Body composition analysis

Body composition was assessed using the in body S10 analyzer. Subjects should fast for 4 h and empty their bladders before lying down for 15 min. After that, they were instructed to stand with arms hanging naturally and abducting by 15 degrees and legs abducting by 30 degrees, maintaining a relaxed and quiet stance. The contact electrodes were disinfected with a 75% alcohol swab. The Thumb and Middle electrodes were connected to the thumb and middle finger of each hand, and the In and Out electrodes were connected to the ankles of each leg. Body composition parameters, such as FFM, SMM, and SLM, are measured using BIA.

### 2.5 Statistical methods

Data were analyzed using SPSS version 26.0. Continuous variables were described using means ± standard deviations (for normally distributed data). Categorical variables were presented as frequencies and percentages. Spearman correlation analysis was employed to explore the relationships between age and both nutritional status and the difference in actual value from normal values in FFM, SMM and SLM (the actual value - the lower limit of normal value). Ordinal multicategory logistic regression was used to analyze the relationship between the exposure factor of age with nutritional status (as an ordinal variable). Meanwhile, multiple linear regression was applied to analyze the relationship between age with differences in FFM, SMM, and SLM (as continuous variables). Statistical significance was set at a *p*-value of less than 0.05.

## 3 Results

### 3.1 Basic information of study objects

This study ultimately enrolled 122 children, consisting of 76 males (62.3%) and 46 females (37.7%), with ages ranging from 2 to 16 years and an average age of 8.71 ± 3.47 years. According to the STAMP nutritional risk screening, 98 children (80.3%) were at moderate nutritional risk, and 24 (19.7%) were at high risk; 100 children (81.9%) had normal BMI-Z/W/H-Z scores, while 22 (18.1%) suffered from wasting. Normally H/A-Z scores were observed in 80 children (65.6%), showing 42 children (34.4%) experiencing stunted growth. The body composition indicators for FFM, SLM, and SMM were 19.2 ± 5.8 kg, 18 ± 5.4 kg, and 9.2 ± 3.4 kg, respectively. The wasting and stunting among the 122 children were divided into five levels: 1, 0, −1, −2, and-3 (with no levels 2 or 3), as shown in Table 1.

**Table 1:** Baseline data for TDT children (*n* = 122).

Item	*n*	Percentage
Age (years, $\bar{x\;}\pm s$ )	8.71 ± 3.47	
Gender		
Male	76	62.3%
Female	46	37.7%
BMI (kg/㎡, $\bar{x\;}\pm s$ )	15.3 ± 1.57	
STAMP		
0–1 points	0	0%
2–3 points	98	80.3%
≥4 points	24	19.7%
BMI-Z /W/H-Z score		
Normal	100	81.9%
Wasting	22	18.1%
H/A-Z score		
Normal	80	65.6%
Stunting	42	34.4%
Body composition parameters		
FFM (kg)	19.2 ± 5.8	
SLM (kg)	18 ± 5.4	
SMM (kg)	9.2 ± 3.4	

### 3.2 Correlation analysis between age and wasting, stunting, and muscle indices

**Figure 1:**
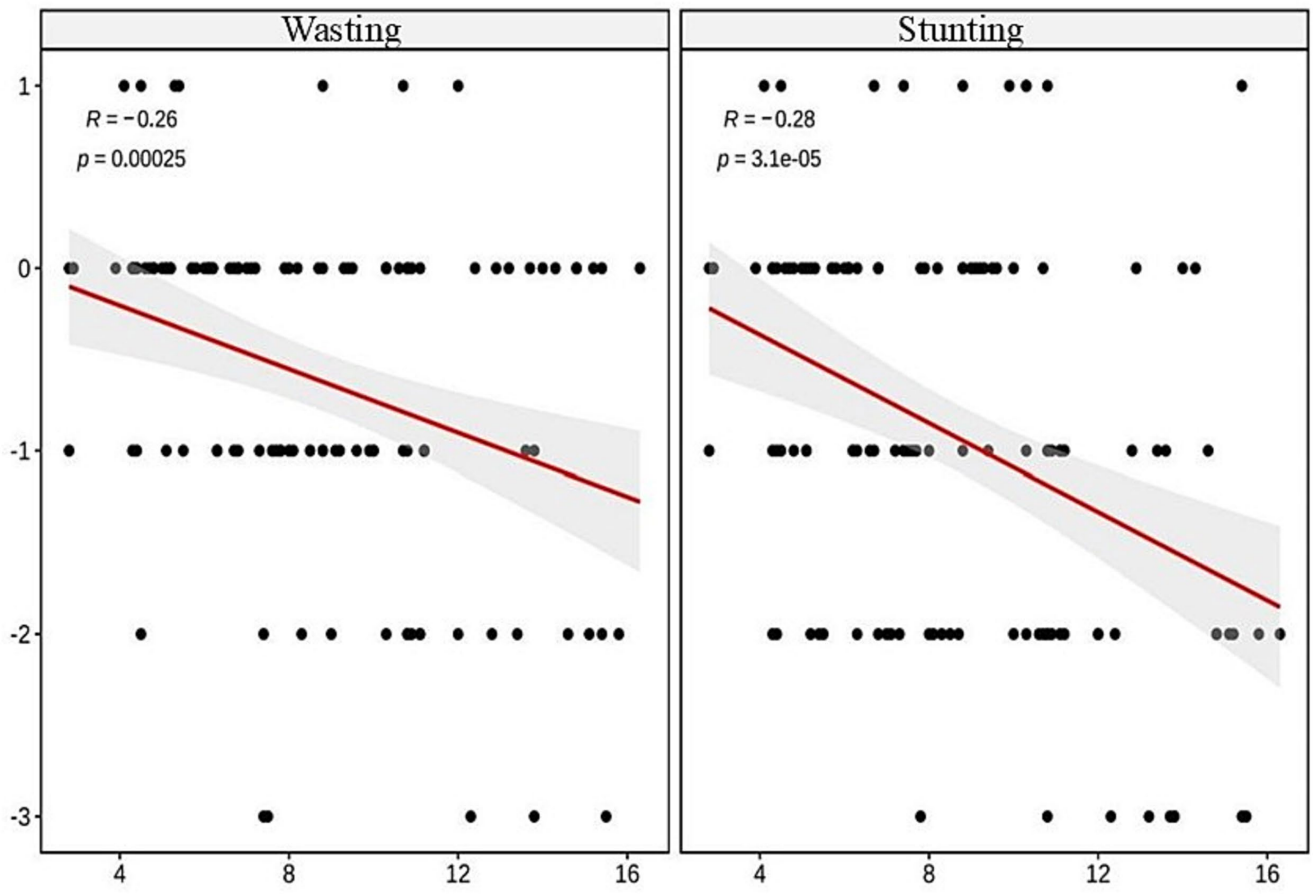
Analysis of the correlation between age and both wasting and stunting.

**Figure 2:**
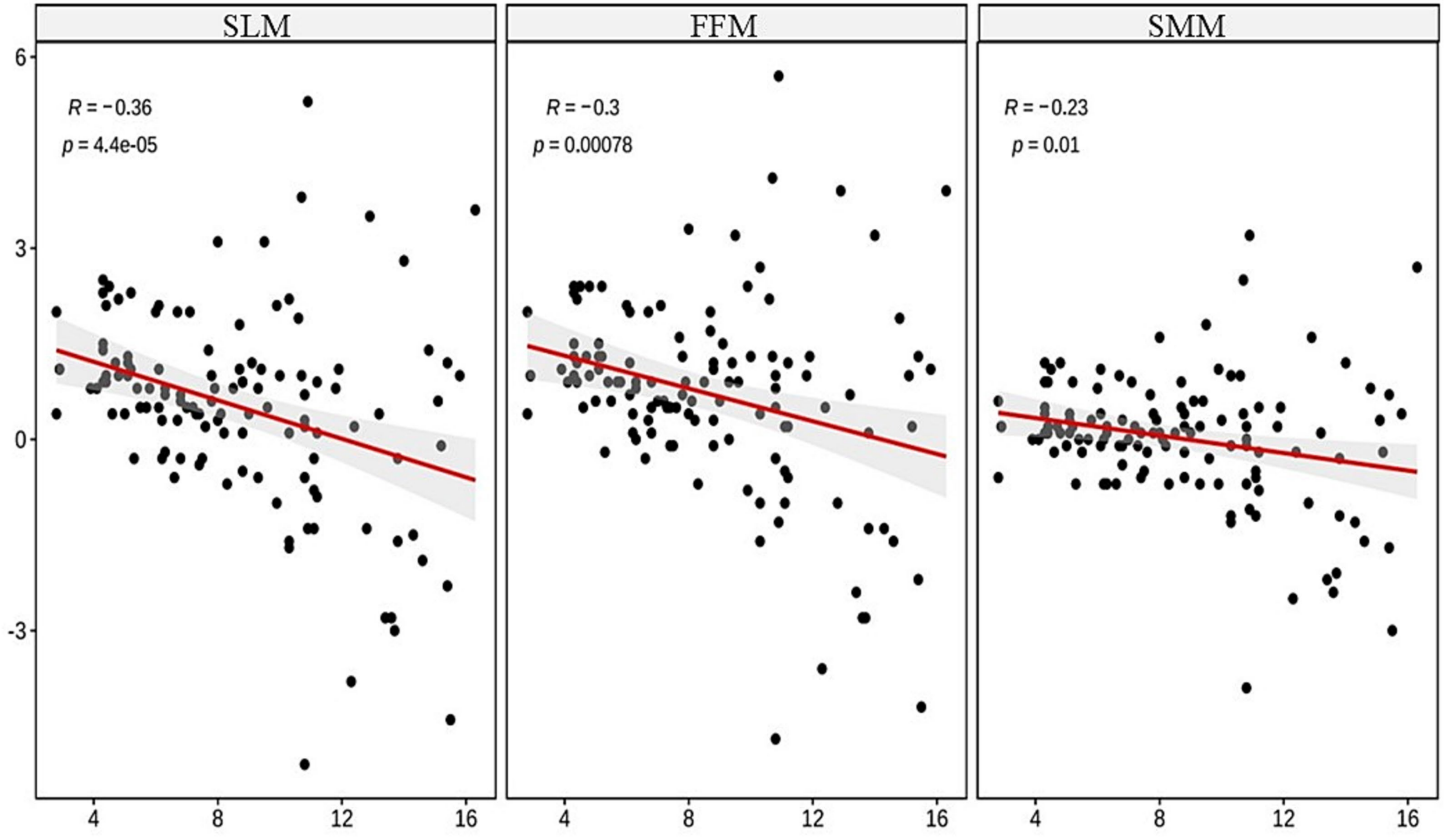
Analysis of the correlation between age and muscle loss.

The Spearman correlation analysis revealed significant negative correlations between age and wasting (*r* = −0.26, *p* < 0.001), stunting (*r* = −0.28, *p* < 0.001), FFM (*r* = −0.3, *p* < 0.001), SMM (*r* = −0.23, *p* < 0.01), and SLM (*r* = −0.36, *p* < 0.001), showing statistical significance, as illustrated in Figures 1, 2.

### 3.3 Regression analysis of age with wasting, stunting, muscle loss

The ordinal multicategory logistic regression analysis indicated a highly significant relationship between age and wasting and stunting in TDT children (both *p* < 0.001), as outlined in Table 2. The multiple linear regression analysis on the effects of age on FFM, SMM, and SLM showed significant relationships between age and the differences (the actual value minus the lower limit of normal value) in these indices (all *p* < 0.01), as presented in Table 3. As age increased, the children were more prone to wasting, stunting and muscle decreasing, suggesting that age is an independent predictor of wasting, stunting, and decreased muscle mass in TDT children, as detailed in Table 3.

**Table 2:** Logistic regression analysis on relationship between age and wasting and stunting in patients with severe TDT.

Independent variable	Dependent variable	β	Z	*p* value
Age	Wasting	−0.17892	−3.35	0.000808
	Stunting	−0.21998	−4.275	<0.001

Wasting and stunting were evaluated as five-level ordinal variables: 1, 0, −1, −2, and-3 (without levels 2 and 3).

**Table 3:** Linear regression analysis on relationship between age and muscle loss in patients with severe TDT.

Independent variable	Dependent variable	*B* value	Standard error	*t* value	*p-* value
Age	SMM - lower limit of SMM	−0.069	0.026	−2.646	<0.01
	FFM lower limit of FFS	−0.129	0.039	−3.276	<0.01
	SLM - lower limit of SLM	−0.151	0.039	−3.873	<0.001

Muscle loss was calculated as the actual values of FFM, SMM, and SLM minus the lower limits of their normal ranges.

## 4 Discussion

This study thoroughly investigated the relationships between age and nutritional status, as well as age and muscle mass, in children with TDT. A significant negative correlation was identified between age and both wasting and stunting, with age emerging as an independent predictor of these conditions. Furthermore, significant associations were observed between age and the loss of FFM, SLM, and SMM, indicating an exacerbation of muscle loss with advancing age. These findings provide crucial insights into the age-related changes in nutritional status and muscle mass among children with TDT.

The multifactorial etiology of progressive wasting, stunting, and muscle loss in TDT children with advancing age can be attributed to several factors. Primarily, chronic anemia affects appetite and increases energy expenditure (25), leading to insufficient energy intake. Additionally, iron overload from transfusions accumulates in the liver, heart, and endocrine organs, causing various complications (1, 26). Furthermore, deficiencies in micronutrients such as zinc and copper, as well as vitamins D and C (27), the use of chelating agents, and dysregulation of the GH-IGF-1 axis due to pituitary iron overload (28–30) are significant contributing factors. Concurrently, protein conversion to glucose through gluconeogenesis to provide energy for the body reduces both muscle and fat mass, resulting in a decreased basal metabolic rate. This cascade effect leads to muscle wasting in children and establishes a negative nitrogen balance in the body (31, 32). This study found that 18.1% of children exhibited wasting and 34.4% showed stunting, which is consistent with previous findings. For example, studies from Pakistan and Iran reported wasting and stunting rates of 40 and 60.7%, and 65%, 41%, respectively (9, 11), reflecting the potential influence of nutritional and socio-economic factors.

Our findings are highly consistent with previous studies across multiple regions. For example, Moiz et al. (11) in Pakistan noted a similar age-related trend, and research from Thailand also observed that the proportions of short stature, underweight, and reduced growth velocity increased with age in TDT children and adolescents (13). Similarly, research from Jordan further confirmed that the proportions of short stature, underweight, and reduced growth velocity all increased with age in TDT children and adolescents (33). These consistent findings across geographical regions emphasize the universal trend of progressive deterioration in nutritional status with age in TDT patients.

Our observation of worsening wasting and stunting with age differs from Mirhosseini et al. (9) who reported age as a positive predictor of nutritional status. However, their identification of puberty as a negative predictor aligns with our findings, as hormonal changes during puberty may exacerbate malnutrition. However, Mirhosseini et al. also identified puberty as a negative predictor of nutritional status, which to some extent supports our observation that increasing age may lead to deterioration in nutritional status. This contradiction may reflect the complex impact of *β*-TM on growth and development, particularly as rapid growth and hormonal changes during puberty may exacerbate the risk of malnutrition.

In this study, a progressive exacerbation of muscle mass loss with increasing age was observed in TDT children. Specifically, the discrepancy in muscle mass between TDT patients and age-matched healthy children widened progressively with increasing age. This finding has significant implications for understanding the nutritional status and long-term health outcomes of TDT patients. However, it is noteworthy that research on the relationship between age and muscle mass in TDT children is relatively scarce, indicating a significant knowledge gap in this area. Existing studies have primarily focused on the phenomenon of muscle loss or impaired muscle function in TDT children, rather than the specific correlation between muscle mass and age (9, 34). While these studies have confirmed muscle-related abnormalities in TDT patients, they fail to delineate the age-dependent progression of muscle mass alterations.

Interestingly, our findings appear to diverge from a 2020 Egyptian study (35), but this discrepancy actually reflects different measurement methods and research focus. The Egyptian study demonstrated significant positive correlations between age and both FFM and muscle mass, reflecting the natural increase in muscle mass during normal child growth and development. In contrast, our study focused on quantifying the magnitude of deviation in muscle mass from age-specific normative values. It was observed that the disparity in muscle mass disparity between TDT children and their healthy counterparts increased with age, suggesting an age-related exacerbation of muscle mass loss. This apparent discrepancy highlights a crucial methodological challenge in pediatric research. During the course of normal growth and development, children typically exhibit an age-associated increase in muscle mass, even in the presence of malnutrition or growth and developmental delays. This physiological phenomenon complicates the accurate assessment of potential muscle loss in TDT children when relying solely on muscle mass measurements. To address this methodological constraint, we implemented an innovative approach. Specifically, we utilized BIA to quantify FFM, SLM, and SMM, subsequently calculating the deviations of these measured parameters from their respective age-specific normative values. This method enabled a more precise identification and quantification of potential muscle mass loss within the context of normal pediatric growth trajectories. Specifically, it enables us to identify potential deviations from normal developmental trajectories, even when overall muscle mass continued to increase. This methodological innovation underscores the importance of using age-specific reference standards, providing a more precise framework for accurately assessing muscle mass and nutritional status in children with chronic diseases such as TDT.

Muscle mass loss is considered a crucial component of malnutrition, with low muscle mass recognized as a core diagnostic criterion for defining adult malnutrition (36), Mager et al.’s study further confirms the relationship between reduced muscle mass in children and poor prognosis (37),Therefore, our findings not only address a significant gap in research regarding age-related changes in muscle mass among TDT children, but also provide a crucial foundation for developing targeted nutritional intervention strategies.

## 5 Conclusion

This study elucidates the complex relationship between age, nutritional status, and muscle mass in children with TDT, highlighting the risk of nutritional deterioration and muscle mass reduction with increasing age. Our findings not only confirm the global trend within the Chinese TDT pediatric population but also emphasize the importance of age-specific assessment methodologies. While the cross-sectional design limits causal inferences, the results provide crucial evidence for developing personalized nutritional management strategies. We recommend establishing interdisciplinary nutritional support teams to regularly assess and adjust nutritional status based on patient age, formulate individualized intervention plans, with particular attention to older children. Concurrently, optimizing transfusion and iron chelation therapy strategies, incorporating appropriate exercise regimens into routine management, and enhancing patient and family education are essential. Future research should focus on longitudinal studies to explore age-specific personalized intervention strategies. Through the implementation of these comprehensive measures, we anticipate improving long-term health outcomes and quality of life for TDT children, effectively mitigating the age-related decline in nutritional status.

## Data Availability

The datasets presented in this study can be found in online repositories. The names of the repository/repositories and accession number(s) can be found at: The datasets generated during and/or analyzed during the current study are not publicly available, but are available from the corresponding author on reasonable request.
